# Expression of tardigrade extremotolerance-associated proteins improves recovery but not acute stress response of human microvascular endothelial cells

**DOI:** 10.1038/s41598-026-57258-y

**Published:** 2026-06-16

**Authors:** William Darch, Janna Soliman, Nicholas C. Higgins, Joshua T. Morgan

**Affiliations:** Department of Bioengineering, University of California, 900 University Ave, Riverside, CA 92521 USA

**Keywords:** Tardigrade, Vascular endothelial cells, Transgenes, Hyperosmotic, Heat Shock, Cell biology, Physiology

## Abstract

Vascular endothelial cells (VECs) play a critical role in tissue and organ homeostasis, wound healing, and immune function. VECs are commonly utilized for in vitro cell culture and in vivo tissue engineering applications which experience intrinsic and extrinsic stressors. Severe stress can overwhelm VECs adaptive stress response mechanisms inducing damage and loss of cell viability. Improving VEC stress tolerance is therefore of increasing interest. In this study we assess the transgenic expression of tardigrade extremotolerance-associated proteins (TEAPs), specifically CAHS3, MAHS, LEAM, and Dsup for improved VEC tolerance to heat shock and hyperosmotic stress. In vitro studies of all TEAP-expressing VECs demonstrate up to 45% decrease in population metabolic activity in response to 24 h incubation in hyperosmotic media solution without recovery. Interestingly, acute hyperosmotic stress treatments followed by a 24 h recovery period showed significant increase in population metabolic activity for CAHS3 (186%), Dsup (202%), and LEAM (223%), while MAHS (161%) showed non-significant increases. Similarly, heat shock treatments followed by a recovery period showed significant increases for CAHS3 (198%), Dsup (182%), MAHS (195%), and LEAM (156%). Our results demonstrate that TEAP expression in VECs confer context dependent protective effects with respect to heat shock and hyperosmotic stress, motivating further exploration of TEAPs stress tolerance function and mechanism of action in mammalian cells.

**Supplementary Information:**

The online version contains supplementary material available at https://doi.org/10.1038/s41598-026-57258-y.

## Introduction

Tardigrades are extremotolerant multicellular microscopic animals that can withstand harsh environmental stressors such as desiccation, radiation, and the vacuum of space^1–4^. While incompletely understood, tardigrade stress tolerance is directly related to the expression of tardigrade unique proteins, here termed tardigrade extremotolerance-associated proteins (TEAPs), and silencing of TEAP genes results in the loss of tolerance and the accumulation of damage^2,5^. While numerous TEAPs have been identified, in this work we focus on several key proteins from *Ramazzottius varieornatus* (*R. varieornatus*), specifically cytosolic-abundant heat soluble 3 (CAHS3), mitochondrial-abundant heat soluble (MAHS), late embryogenesis-abundant mitochondrial (LEAM; also known as RvLEAM), and damage suppressor (Dsup)^2,5–9^. CAHS3, MAHS, LEAM, and Dsup are TEAPs that can be classified as intrinsically disordered proteins (IDPs) or possess a high degree of intrinsically disordered regions (IDRs)^2,9–12^. IDPs exist as dynamic conformational ensembles with myriad binding mechanisms (e.g., dynamic complex formation) that further expand their adoptable structures and interaction partners^13^. This inherent property of IDPs has been hypothesized as a mechanism to regulate IDP function^13^, although this is understudied in TEAPs specifically.

CAHS family of proteins predominantly localize to the cytosol and less commonly to the nucleus^7^. RNAi knockdown studies of CAHS gene expression significantly decreased survival of *Hypsibius dujardini* to desiccation stress, highlighting the critical function of CAHS proteins for desiccation tolerance^2^. Engineered yeast and bacteria expressing CAHS proteins showed significant increases in survival to desiccation^2,14^ and biofuels^15^. Furthermore, purified CAHS proteins conferred resistance to desiccation induced deactivation of lactate dehydrogenase enzymes^2^.

LEAM is a mitochondrial LEA protein and MAHS is a mitochondrial heat soluble protein unique to tardigrades^8^. Studies by Tanaka et al. demonstrated transgenic expression of LEAM or MAHS in HEp-2 cells treated with hyperosmotic media enhanced metabolic activity compared to controls, with LEAM conferring higher stress tolerance^8^. Since the pioneering work by Tanaka et al. there have been multiple papers exploring the potential function of LEAM and MAHS proteins for diverse applications. A recent study demonstrated that a truncated form of LEAM termed RvLEAM_short_ enhanced sample quality post cryo-EM plunge freezing^16^. Our recent paper tested stress tolerance (e.g., DMSO, freezing, nutrient deprivation, and injection shear stress) and differentiation potential of transgenic human adipose-derived stem cells (ASCs) stably expressing MAHS protein^17^. Results showed MAHS localized to the mitochondria and provided a significant increase in ASC viability after treatments with different stressors including DMSO, nutrient depletion, and shear stress. Interestingly, ASCs expressing MAHS protein also showed a bias for differentiation into osteogenic rather than adipogenic lineages, while increased glucose supplementation during differentiation partially rescued the adipogenic phenotype^17^.

Dsup is a highly disordered TEAP with a bipartite nuclear localization signal—which drives its import into the nucleus—and a net positive charge that results in colocalization with nuclear DNA^5,6,18,19^. Further, Dsup possesses an HMGN-like region that promotes binding to nucleosomes over free DNA^18^. In the presence of oxidative stress researchers showed that Dsup protected both free DNA and chromatin from ROS induced DNA damage, with greater protection to chromatin^18^. Hashimoto et al. demonstrated that transgenic HEK293 cells expressing Dsup mitigated X-ray and ROS-induced DNA damage, verified by comet assays and γ-H2AX antibody stain^5,6^. In vivo DNA damage studies in mice showed local treatment of oral and rectal tissues with Dsup mRNA polymer-lipid nanoparticles (LNP) conferred a significant decrease in DNA damage signaling in response to ionizing radiation compared to control mRNA polymer-LNP and non-treatment groups^20^. In contrast, recent studies demonstrated that Dsup exhibits toxicity in primary neurons promoting increased DNA damage and altered organization of chromatin^21^. The significant difference in genotoxicity between immortalized cancer and primary cell lines expressing Dsup motivates further studies of TEAPs expressed in diverse cell types.

Due to these unique properties, TEAP’s possess potential for diverse applications in bioengineering (e.g., medicine, biomaterials, and agriculture). Experimental studies on the transgenic expression of TEAPs in human cells have highlighted therapeutic potential, however, deleterious effects have been documented in a cell-type dependent context, motivating further study of these proteins in diverse cell types. In this study, we are primarily concerned with TEAP expression in vascular endothelial cells (VECs). VECs play a critical role in tissue and organ homeostasis, wound healing, and immune function^22–25^. Additionally, development of stable vasculature is considered a key challenge in tissue engineering and the development of organotypic models^26–35^. In both native and transplanted tissues, cellular stress dysregulates homeostatic mechanisms, damages macromolecules, and impairs function. More specifically, VEC damage drives inflammation, senescence, dysfunction, and apoptosis^36,37^. In native tissue, this is associated with the development and progression of multiple diseases (e.g., cardiovascular, neurodegenerative, diabetes)^36–39^; in graft transplants, this is associated with graft failure^40–46^.

Here we demonstrate that *R. varieornatus* TEAPs (i.e., CAHS3, MAHS, LEAM, and Dsup) can be stably expressed in immortalized human microvascular endothelial cells (HMEC1) and retain proliferative activity. As representative stressors we focus on hyperosmotic stress and heat shock; population health is quantified using population-level metabolic activity (MTT). We demonstrate HMEC1 cells expressing TEAPs have increased stress tolerance. Specifically, we show TEAPs decreased metabolic activity compared to control AcGFP1-expressing cells immediately post-hyperosmotic stress but that TEAP expression increased metabolic activity after a 24 h recovery compared to control cells. We further show that TEAPs net increase HMEC1 metabolic activity after recovery from heat shock in comparison to AcGFP1-expressing controls. These findings further demonstrate the potential for TEAP expression to boost stress tolerance in specific human cell types.

## Materials and methods

### Routine cell culture

Immortalized human microvascular endothelial cells (HMEC1; American Type Culture Collection, Manassas, VA) modified with TEAPs or control genes were routinely cultured in MCDB131 without L-Glutamine (CM034-050; GenDEPOT, Baker, TX) and supplemented with [10 ng/mL] Epidermal Growth Factor (AF-100-15; Peprotech, Cranbury, NJ), [1 µg/mL] hydrocortisone (A1629203; ThermoFisher Scientific, Waltham, MA), [10 mM] L- Glutamine (ThermoFisher Scientific), 10% fetal bovine serum (FBS; ThermoFisher Scientific), 1% penicillin-streptomycin (P/S; Mediatech, Manassas, VA), [1 µg/mL] amphotericin B (ThermoFisher Scientific). Cells were consistently passaged at ~ 80% confluence and all experiments were conducted with cells below twenty-five passages post lentiviral transduction. HEK293TN cells (LV900A-1; System Biosciences, LLC Palo Alto, CA) were maintained in DMEM with [4.5 g/L] glucose, L-glutamine & sodium pyruvate (10-013-CM; Mediatech), supplemented with 10% FBS, 1% P/S, and [1 µg/mL] amphotericin B. All cultures were maintained at 37 °C and 5% CO_2_.

### Cloning

AcGFP1-N1 [AcGFP1-N1 was a gift from Michael Davidson (Addgene plasmid # 54705 ; http://n2t.net/addgene:54705 ; RRID: Addgene_54705)], AcGFP1-N1-CAHS3 [pAcGFP1-N1-CAHS3 was a gift from Takekazu Kunieda (Addgene plasmid # 90031 ; http://n2t.net/addgene:90031 ; RRID: Addgene_90031)], AcGFP1-N1-Dsup [pAcGFP1-N1-Dsup was a gift from Takekazu Kunieda (Addgene plasmid # 90020 ; http://n2t.net/addgene:90020 ; RRID: Addgene_90020)], AcGFP1-N1-MAHS [pAcGFP1-N1-MAHS was a gift from Takekazu Kunieda (Addgene plasmid # 90034 ; http://n2t.net/addgene:90034 ; RRID: Addgene_90034)], and AcGFP1-N1-RvLEAM [pAcGFP1-N1-RvLEAM was a gift from Takekazu Kunieda (Addgene plasmid # 90035 ; http://n2t.net/addgene:90035 ; RRID: Addgene_90035)] fusions were independently cloned into the 2nd-generation lentiviral transfer vector (pCDH-CMV-MCS-EF1α-Puro; Systems Biosciences, Palo Alto, CA).

In all cases, double restriction digests were performed (EcoRI and NotI for AcGFP1-N1, AcGFP1-N1-Dsup; NheI and NotI for AcGFP1-N1-CAHS3, AcGFP1-N1-MAHS, and AcGFP1-N1-RvLEAM). Restriction products were purified using horizontal agarose gel electrophoresis followed by E.Z.N.A. gel extraction kit cleanup (Omega Bio-tek; Norcross, GA). The genes of interest were separately ligated into pCDH-CMV-MCS-EF1α-Puro using T4 DNA ligase (New England Biolabs, Ipswich, MA). Ligation products were independently transformed into chemically competent DH5α (Mix & Go! competent cells; Zymo research, Irvine, CA). Transformed cells were plated on LB agar with ampicillin and cultured overnight at 37 °C. Bacterial colonies were selected for inoculation of liquid LB broth and cultured overnight at 37 °C, followed by plasmid DNA purification (E.Z.N.A. plasmid DNA mini kit I; Omega Bio-tek). Purified lentiviral transfer plasmids pCDH-CMV-AcGFP1-N1-EF1α-Puro, pCDH-CMV-AcGFP1-N1-CAHS3-EF1α-Puro, pCDH-CMV-AcGFP1-N1-Dsup-EF1α-Puro, pCDH-CMV-AcGFP1-N1-MAHS-EF1α-Puro, and pCDH-CMV-AcGFP1-N1-RvLEAM-EF1α-Puro sequences were verified with Sanger sequencing (Retrogen Inc., San Diego, CA).

### Lentiviral transduction and selection

HEK293TN cells were cultured to 80% confluence and placed in fresh media prior to triple transfection with Endofectin Max (EF013; GeneCopoeia, Rockville, MD), packaging plasmid psPAX2 (Addgene # 12260), envelope plasmid pMD2.G (Addgene # 12259), and either lentiviral transfer vector pCDH-CMV-AcGFP1-N1-EF1α-Puro, pCDH-CMV-AcGFP1-N1-CAHS3-EF1α-Puro, pCDH-CMV-AcGFP1-N1-Dsup-EF1α-Puro, pCDH-CMV-AcGFP1-N1-MAHS-EF1α-Puro, or pCDH-CMV-AcGFP1-N1-RvLEAM-EF1α-Puro. Viral supernatant was collected at 48 h, centrifuged at 1000 × *g* for 10 min and 0.45 μm filtered prior to addition to HMEC1 cells. Transduced cells were selected with [0.2 µg/mL] puromycin for four weeks to obtain a stably expressing population confirmed via fluorescence microscopy. Further, we routinely checked transgene expression via fluorescence microscopy prior to experimentation. We consistently observed proliferation and normal morphology in all transgenic cultures for the duration of our studies.

### Subcellular localization of TEAPs

All transgenic cell lines were stained with 500 nM Hoechst 33342 (AAT Bioquest, Pleasanton, CA) in PBS; cells were incubated for 20 min at 37 °C followed by three wash steps with PBS. Cells were immediately stained with [100 nM] Tetramethylrhodamine, Ethyl Ester (TMRE T669, ThermoFisher Scientific) in PBS. Cells were then incubated for 15 min at 37 °C and 5% CO2. Samples were imaged with a DFC9000GT sCMOS camera on a DMi8 inverted microscope (Leica Microsystems, Chicago, IL).

### Transgenic HMEC1 proliferation

Transgenic HMEC1 cell lines (AcGFP1, CAHS3, Dsup, MAHS, and LEAM) were seeded at a cell density of 500,000 cells per 90 mm plate in routine culture media. Cells were refed with fresh media every three days. We captured tilescans (36 tiles) of each transgenic cell line over a five-day period at 24-, 72-, and 120-hour timepoints.

### Hyperosmotic stress

Experiments were conducted in 24-well plates. Transgenic HMEC1 cell lines (AcGFP1, CAHS3, Dsup, MAHS, and LEAM) were seeded at an initial cell density of 100,000 cells per well in routine culture media and incubated for 24 h at 37 °C and 5% CO_2_. After 24 h, controls were replaced with fresh media and experimental groups were replaced with media supplemented with [700 mOsM] xylitol; prior studies with xylitol have demonstrated its utility for hyperosmotic stress induction^47^. Cells were incubated for 24 h at 37 °C and 5% CO_2_. No recovery hyperosmotic stress experiments both treatment and control groups were refed with fresh media supplemented with MTT. Recovery hyperosmotic stress experiments both treatment and control groups were refed with fresh media and allowed to recover at 37 °C and 5% CO_2_ for 24 h, then both treatment and control groups were refed with fresh media supplemented with MTT.

### Heat Shock

Experiments were conducted in 24-well plates. Transgenic HMEC1 cell lines (AcGFP1, CAHS3, Dsup, MAHS, and LEAM) were seeded at an initial cell density of 100,000 cells per well in routine culture media and incubated for 24 h at 37 °C and 5% CO_2_. After 24 h, culture media was replaced with fresh media and cells were incubated for 1 h at 37 °C and 5% CO_2_. Experimental heat shock treatment group was then transferred to an incubator at 44 °C and 5% CO_2_ for a duration of 2 h. Post heat shock treatment both control and experimental groups were refed with fresh media and allowed to recover at 37 °C and 5% CO_2_ for 24 h. Post-recovery cell metabolic activity was measured utilizing MTT assay.

### Metabolic activity (MTT) assay

An MTT assay was used to assess metabolic activity of the population. MTT is often used to measure cell viability of a population^48^, and functions primarily through reductive activity mediated by NAD(P)H and glycolytic enzymes in active cells^49^. Increased signal can be due to increased cell number, increased metabolic activity, or other factors. Briefly, a stock solution of thiazolyl blue tetrazolium bromide (MTT) was prepared at 5 mg/mL in PBS, vortexed, and filter sterilized. For the assay, MTT was diluted 1:10 in fresh media for a final working concentration of [0.5 mg/mL] MTT and added to experimental and control groups. Cells were then incubated 12 h at 37 °C and 5% CO_2_. Wells were gently aspirated and allowed to dry for 20 min; formazan crystal was solubilized with 400 µL of DMSO and mixed via pipetting. Plates were then covered in tin foil and incubated in a benchtop orbital shaker (Thermo Scientific MaxQ 4450) at 125 rpm and 37 °C for 15 min. Formazan solution was then transferred into a 96-well plate with technical triplicates of 100 µL for quantitation using 570 absorbance (SpectraMax M2, Molecular Devices, San Jose, CA).

### Statistics

All measurements were normalized to within genotype controls that were not exposed to stress. Analysis of variance (ANOVA) with replicates as a fixed block effect and transgene as a treatment effect was used on log normalized data. Post hoc comparison between TEAPs and AcGFP1 control group were performed using Dunnett’s test with AcGFP1 serving as control group. All statistical tests and data visualization were performed with MATLAB. Datasets utilized to produce graphical representations and statistical analyses are included in supplementary information.

## Results

### Localization of AcGFP1-tagged TEAPs in HMEC1 cells

**Fig. 1:**
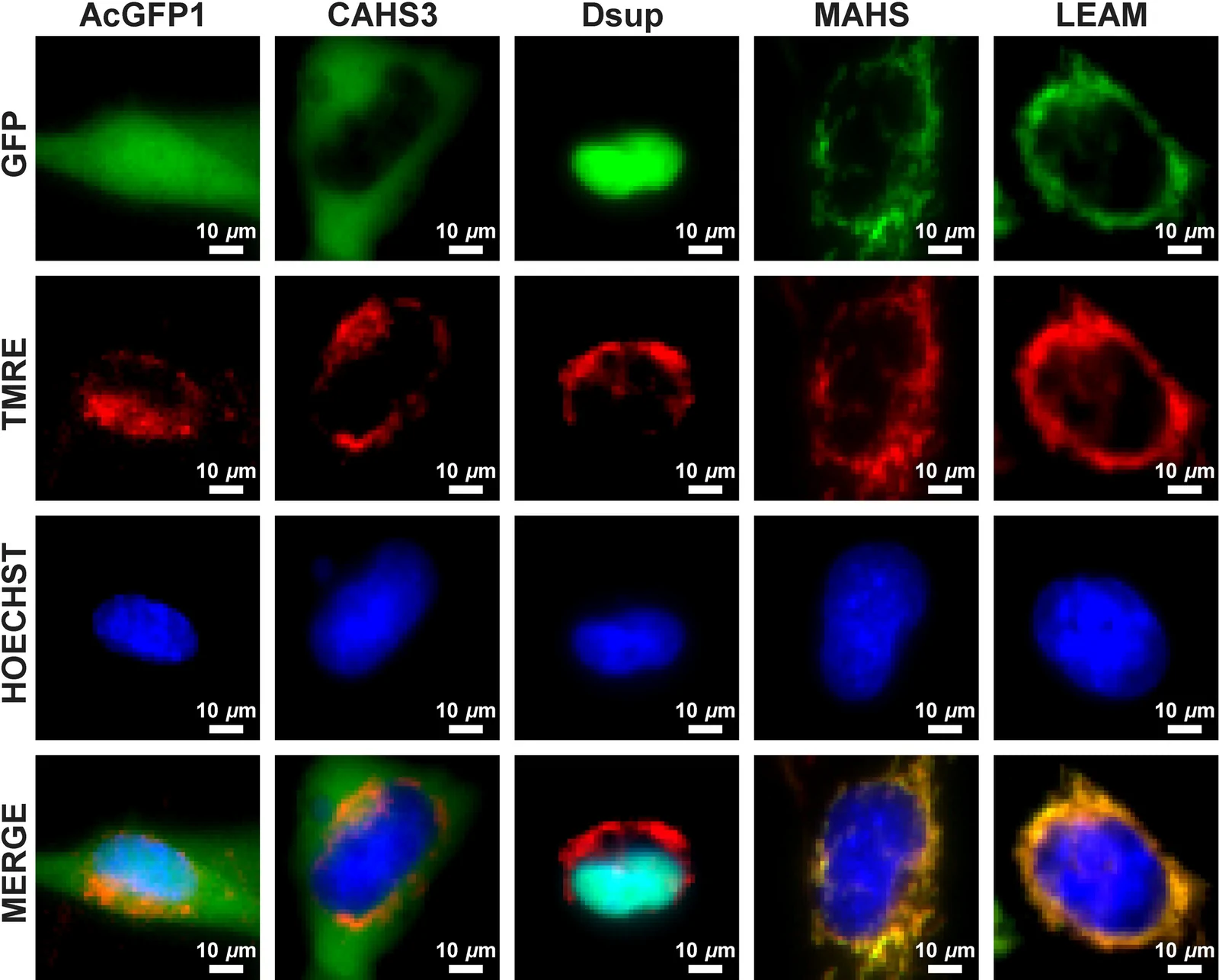
Expression of AcGFP1 (control) and AcGFP1-tagged TEAPs (CAHS3, Dsup, MAHS, and LEAM) in human microvascular endothelial cells. Transgenic HMEC1 expressing AcGFP1-tagged TEAPs were stained live with the cell permeant cationic mitochondrial dye TMRE (red) and nuclear DNA counterstain Hoechst (blue). AcGFP1 is expressed nonspecifically throughout the cell, CAHS3 localizes to the cytosol, Dsup localizes to the nucleus, and both MAHS and LEAM localize to the mitochondria.

Transgenic HMEC1 cells expressing AcGFP1 (control) and AcGFP1-tagged TEAPs (CAHS3, Dsup, MAHS, or LEAM) were stained with TMRE (mitochondrial membrane) and Hoechst (nuclear) dyes to qualitatively assess localization of TEAPs with fluorescence microscopy imaging (Fig. 1). Qualitatively, we observed AcGFP1 (control) nonspecifically diffused throughout the cell, CAHS3 localized to the cytosol, Dsup colocalized with DNA in the nucleus, and both MAHS and LEAM localized to the mitochondria. These results were consistent across the cell population, larger fields-of-view shown in Figure S1. Quantitative studies of localization were not performed.

### Transgenic HMEC1 cells express TEAPs and proliferate with normal morphologies

**Fig. 2:**
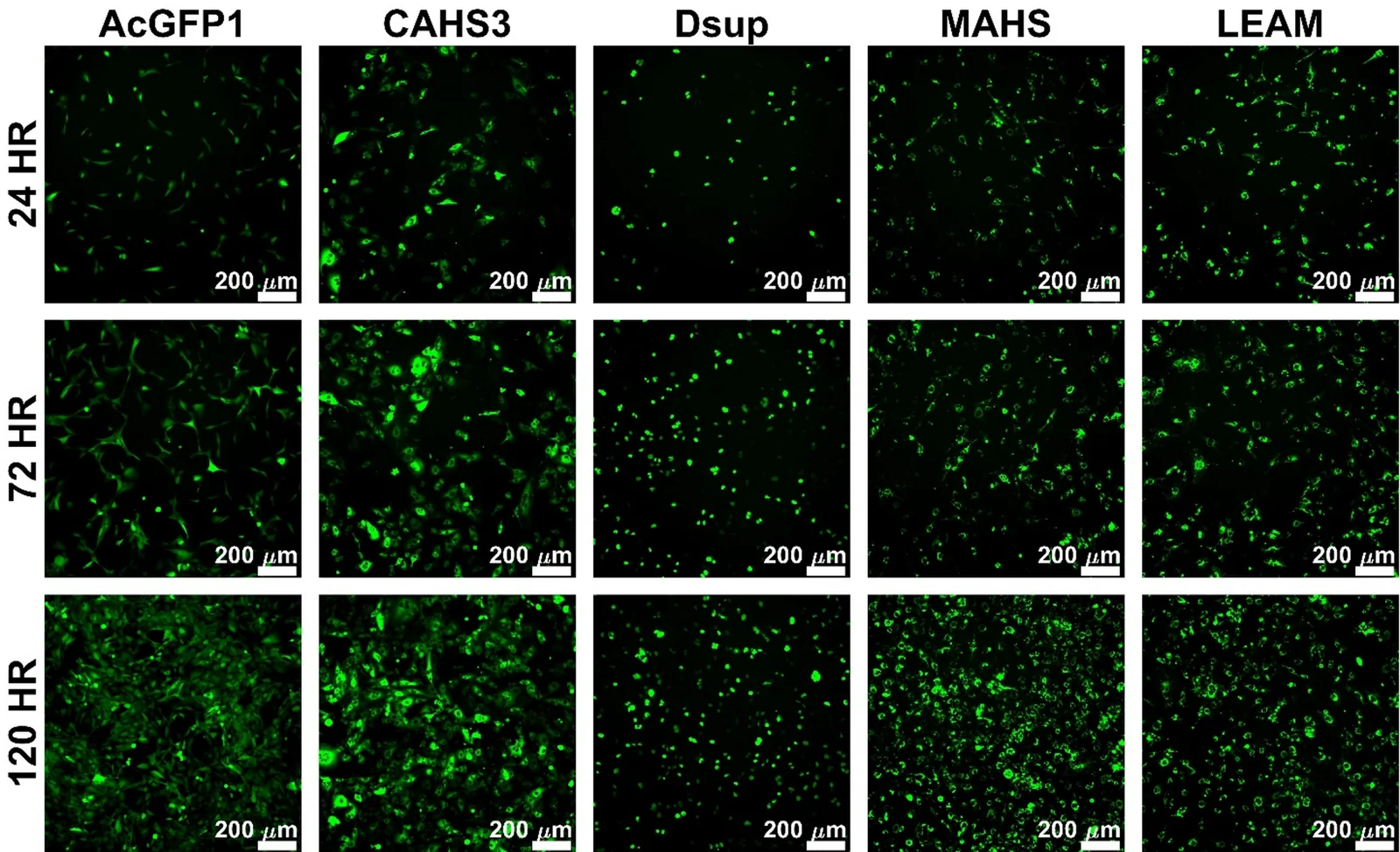
Transgenic HMEC1 stably express TEAPs and proliferate with normal morphology under routine cell culture conditions. Transgenic HMEC1 cells were seeded in a 90 mm tissue culture plate at 500,000 cells in a total volume of 9 mL of fresh media and maintained following routine culture conditions. Cells were imaged at 24, 72, and 120 h timepoints to assess growth and morphology. Transgenic HMEC1 cells expressing TEAPs proliferated until confluent and displayed morphologies consistent with parental HMEC1 cells.

We assessed proliferative activity of our stable transgenic HMEC1 population by monitoring cell growth. Each transgenic cell line was seeded at a low density of 500,000 cells per 90 mm plate and allowed to proliferate over five days of culture. Cell density increased continuously to confluence in all genotypes (Fig. 2), indicating that the transgenic cells can be routinely cultured without gross defects in cell proliferation or survival. Quantitative studies of proliferation rate or mitotic index were not performed.

### TEAPs decrease population metabolic activity immediately post hyperosmotic stress

**Fig. 3:**
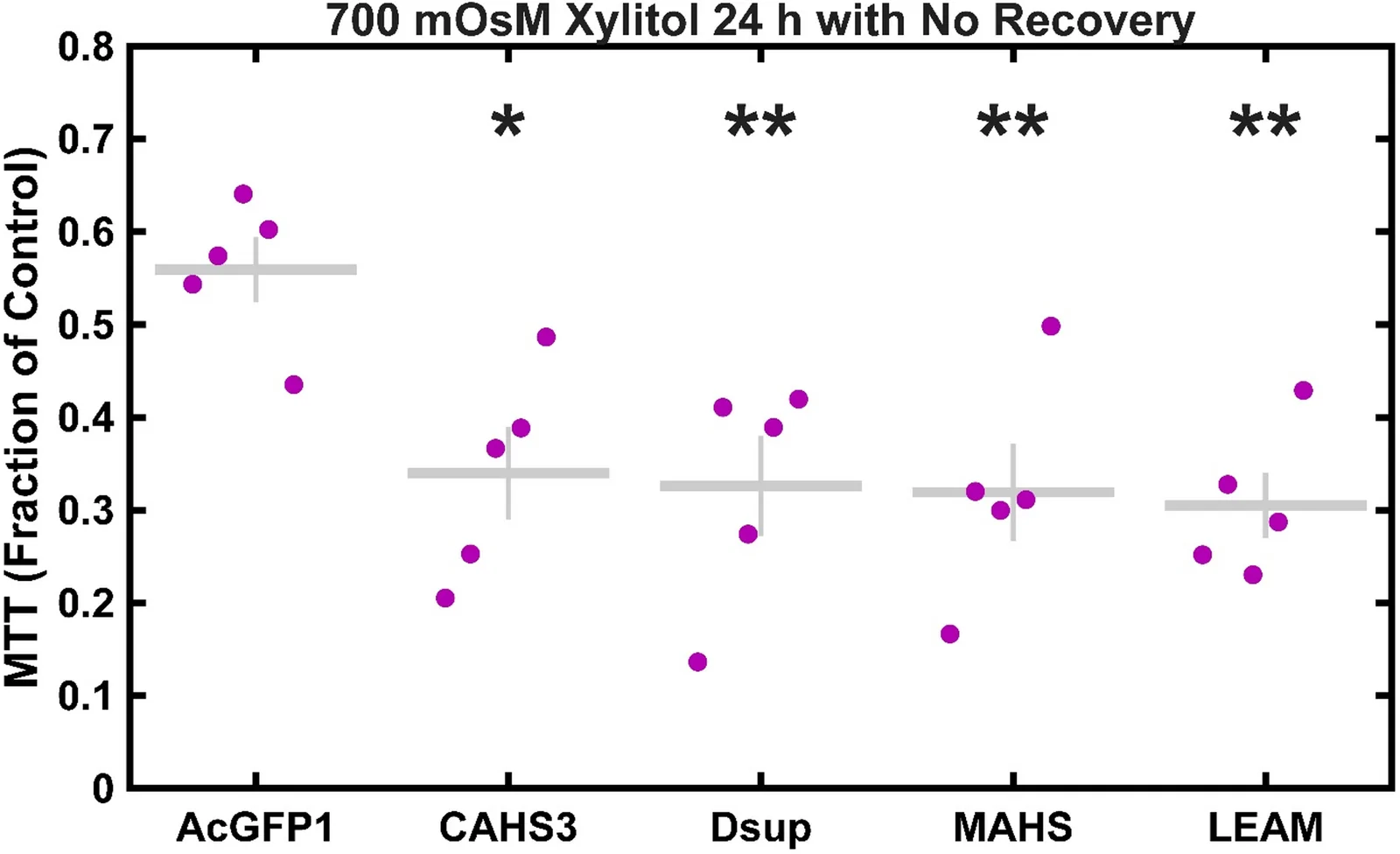
Transgenic HMEC1 cells expressing TEAPs treated with hyperosmotic stress (i.e., 700 mOsM xylitol for 24 h) showed decreased metabolic activity immediately post stress quantified by MTT. All measurements were normalized to within genotype controls that were treated with normal media (no xylitol). Statistical significance determined by ANOVA followed by Dunnett’s post-hoc test using AcGFP1 expressing cells as control. *n* = 5 (* *p* < 0.05 Dunnett’s; ** *p* < 0.01 Dunnett’s).

Having established that TEAPs localize as expected in HMEC1 cells and do not grossly disrupt cell proliferation, we wished to test the effect of TEAP expression on HMEC1 tolerance of hyperosmotic stress. Transgenic HMEC1 cells treated with hyperosmotic media solutions (700 mOsM xylitol) for a duration of 24 h followed by immediate assessment of population-level metabolic activity (i.e., no recovery period) showed a significant reduction in CAHS3 (0.34 ± 0.05; *p* = 0.0157), Dsup (0.33 ± 0.05; *p* = 0.063), MAHS (0.32 ± 0.05; *p* = 0.0062), and LEAM (0.31 ± 0.04; *p* = 0.0053) expressing cells compared to AcGFP1 controls (0.56 ± 0.03). These data are consistent with a loss of population-level metabolic activity immediately after hyperosmotic challenge due to cell loss or other mechanisms (Fig. 3).

### TEAPs protect population metabolic activity after recovery from acute hyperosmotic stress

**Fig. 4:**
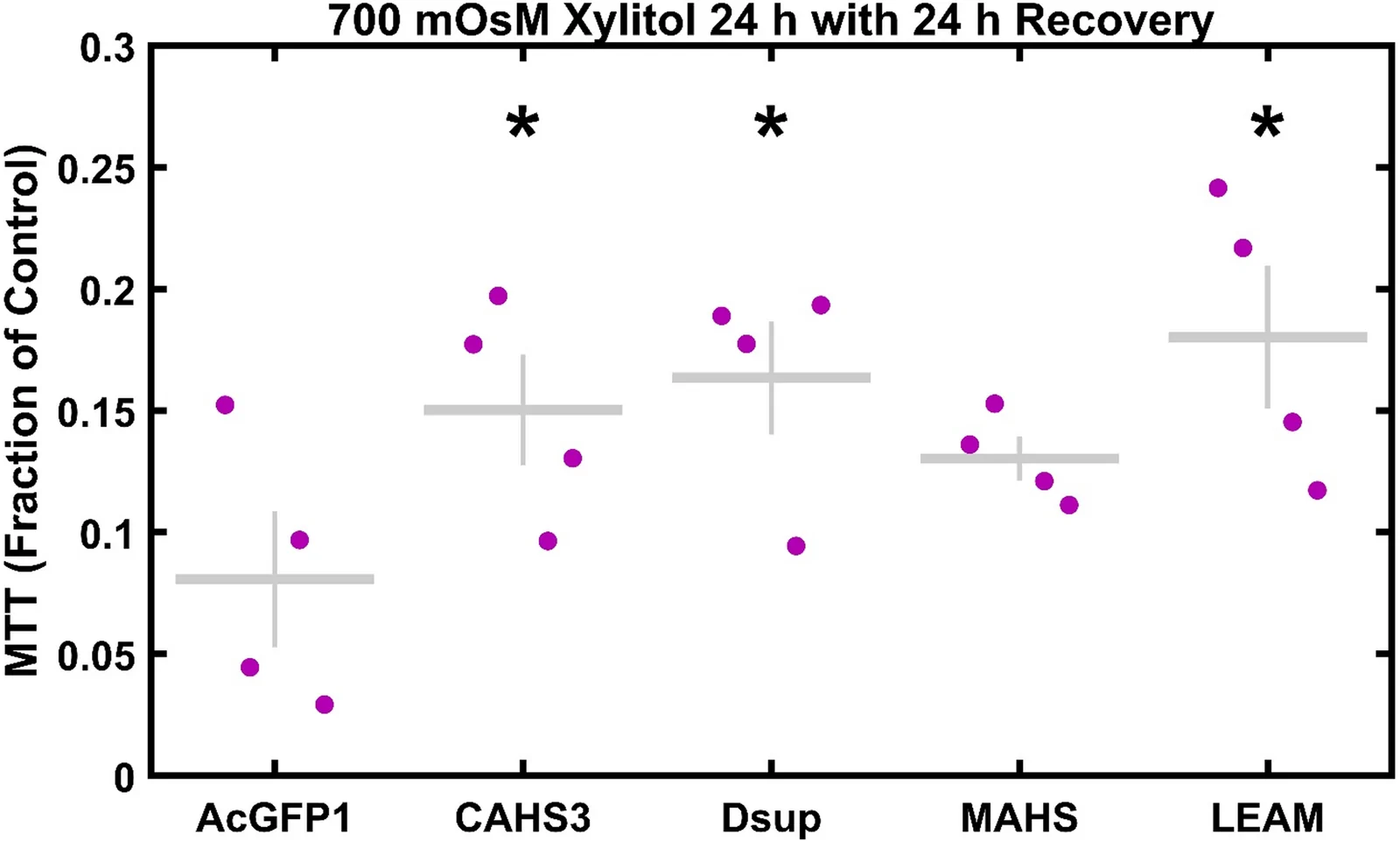
Transgenic HMEC1 cells expressing TEAPs treated with hyperosmotic stress (i.e., 700 mOsM xylitol for 24 h) showed increased metabolic activity immediately post recovery quantified by MTT. All measurements were normalized to within genotype controls that were treated with normal media (no xylitol). Statistical significance determined by ANOVA followed by Dunnett’s post-hoc test using AcGFP1 expressing cells as control. *n* = 4 (* *p* < 0.05 Dunnett’s).

We further wished to assess if there was a longer term protective effect. Transgenic HMEC1 cells treated with hyperosmotic media solutions (700 mOsM Xylitol) for a duration of 24 h, similar to above, but then given a recovery period of 24 h in fresh media followed by assessment of metabolic activity. Here CAHS3 (0.15 ± 0.02; *p* = 0.042), Dsup (0.16 ± 0.02; *p* = 0.024), and LEAM (0.18 ± 0.03; *p* = 0.013) expressing cells showed a significant increase in metabolic activity compared to AcGFP1 controls (0.08 ± 0.03), with a non-significant increase for MAHS (0.13 ± 0.09; *p* = 0.088) expressing cells (Fig. 4). These data are consistent with protection of population-level metabolic activity compared to control, indicating increased cell survival or protection of metabolic function.

### TEAPs protect population metabolic activity post heat shock and recovery

**Fig. 5:**
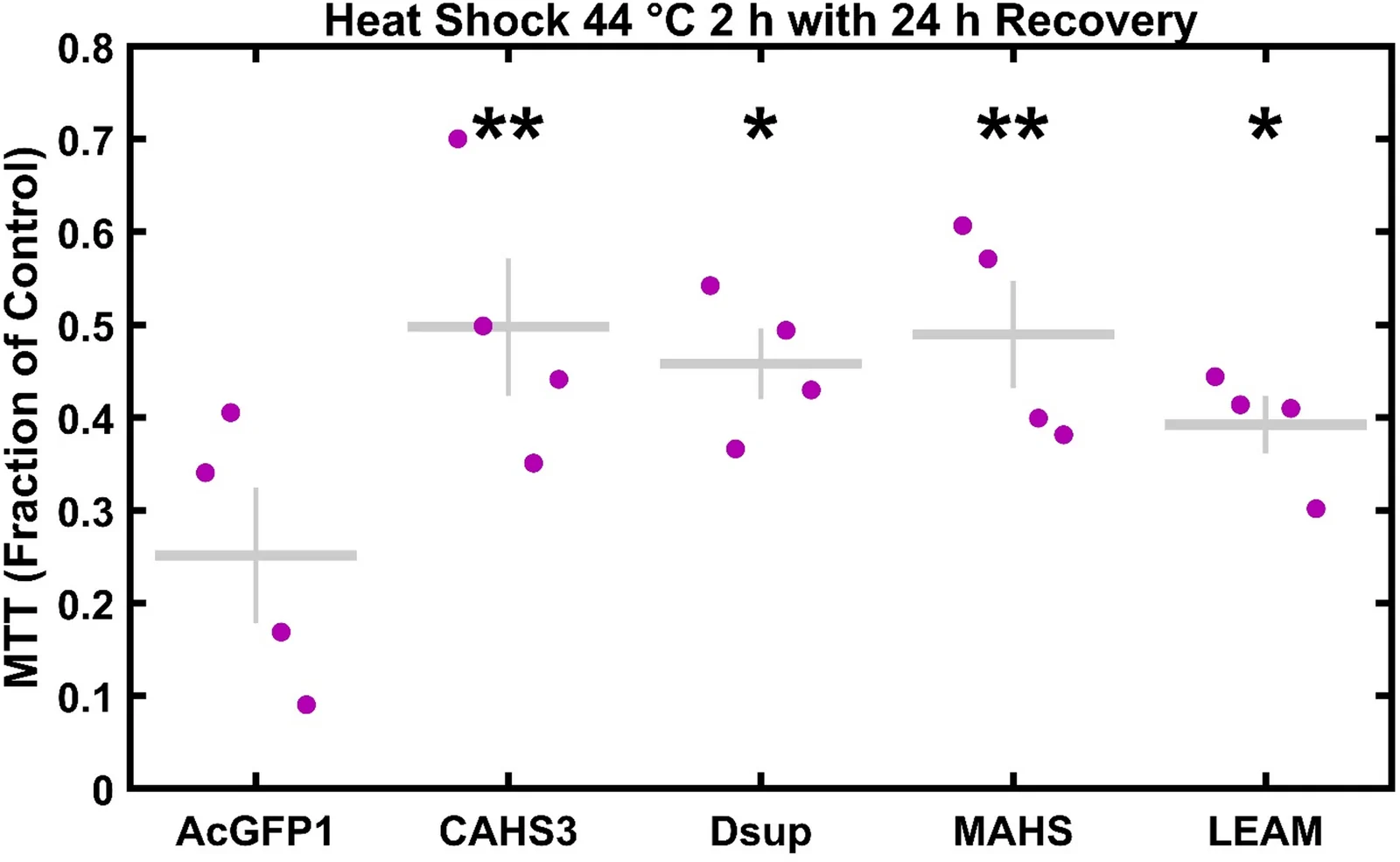
Transgenic HMEC1 cells expressing TEAPs treated with heat shock stress (i.e., 44 °C for 2 h) showed increased metabolic activity immediately post recovery quantified by MTT. All measurements were normalized to within genotype controls that were not heat shocked. Statistical significance determined by ANOVA followed by Dunnett’s post-hoc test using AcGFP1 expressing cells as control. *n* = 4 (* *p* < 0.05 Dunnett’s; ** *p* < 0.01 Dunnett’s).

As another representative stressor, we wished to test the protective effect of TEAPs to heat shock. Transgenic HMEC1 cells were heat shocked (44 °C) for a duration of 2 h then given a recovery period of 24 h in fresh media followed by assessment of metabolic activity. Expression of CAHS3 (0.50 ± 0.07; *p* = 0.0068), Dsup (0.46 ± 0.04; *p* = 0.0117), MAHS (0.49 ± 0.06; *p* = 0.0072), and LEAM (0.39 ± 0.03; *p* = 0.0442) demonstrated a significant increase compared to AcGFP1 controls (0.25 ± 0.07) as shown in (Fig. 5). These data are consistent with protection of population-level metabolic activity compared to control, indicating increased cell survival or protection of metabolic function.

## Discussion

TEAPs have been previously demonstrated to have protective function in human cells^5,8,17,50,51^, although toxic effects have been observed in rat neurons^21^, demonstrating a need for careful assessment within specific cell contexts. While data is overall limited, prior studies suggest that the protective (or toxic) effects of TEAP expression partially depend on cell type and the specifics of stress application, motivating studies in specific cell types with diverse stress conditions. Further, prior studies in mammalian cells have commonly utilized transient transfection which may not reveal issues with stable expression. Here, we demonstrate stable expression of several TEAPs in a human model of VECs, HMEC1 and demonstrate context dependent protective effects. We chose hyperosmotic and heat shock as generic stressors given their broadly activating stress responses^52–54^. Briefly, increases in extracellular osmolarity (hyperosmotic stress) drives intracellular water efflux promoting the rapid reduction in cell volume, concentration of ions, and crowding of macromolecules which leads to loss of cell viability if homeostasis is not reestablished^55,56^. High concentrations of extracellular solutes (e.g., sorbitol, NaCl) have been shown to: suppress antioxidant enzymes^57^; impair multiple DNA damage repair proteins^58^; increase ROS production^57,59^; damage biomolecules^57,58,60^; trigger ER stress^61^; induce mitochondrial dysfunction, depolarization, and permeability^59,62^; and drive apoptosis^59,62–64^. Similarly, severe or prolonged heat shock overwhelms stress responses mechanisms (e.g., heat shock proteins) inducing myriad of deleterious downstream effects activating cell death pathways^53,54,65^.

In contrast to prior results expressing LEAM and MAHS in HEp-2 cells^8^, we observed reduced metabolic activity compared to controls immediately following osmotic challenge (Fig. 3). We observed similar reductions with CAHS3 and Dsup (Fig. 3). These data are consistent with TEAPs having cell type and context specific effects; Dsup studies by Escarcega et al., highlighted the cell type dependent toxicity of TEAP expression in primary neurons compared to HEK293 cells^21^. Interestingly, we also observed significant protection in metabolic activity in CAHS3, Dsup, and LEAM expressing cells following a 24-hour recovery in comparison to control cells, consistent with a protective effect (Fig. 4). We similarly observed significant protection after heat shock and recovery for CAHS3, Dsup, MAHS, and LEAM demonstrating that these molecules are protective against multiple types of stress (Fig. 5).

Of special interest in these data are the apparent cytotoxicity during the acute hyperosmolarity followed by improved recovery. These responses need not be mutually exclusive. Cells undergoing hyperosmotic stress immediately undergo cell shrinkage and several negative effects, including DNA double strand breaks, cell cycle arrest, and apoptosis^56,58,62–64^. This damage is paralleled by active cytoskeletal and transport processes to increase cell volume, termed regulatory volume increase (RVI)^52,56^. It has been suggested that apoptosis is associated with insufficient or impaired RVI^56^. For surviving cells, early volume responses are followed by changes in osmolyte transport and synthesis, as well as heat shock protein (HSP) expression^52,56^. The HSP family has a wide range of pro-survival mechanisms, however one of the most important is chaperone function; that is, stabilization of cellular proteins during stress^66,67^. While TEAP function is incompletely understood, it has been speculated that they act as chaperones that stabilize protein, membrane, and cell structure during desiccation in the native tardigrade, for example by preventing protein denaturation and membrane fusion^2,9,51,68–71^. It is possible that TEAPs expressed in HMEC1s inhibit or disrupt the early response to hyperosmolarity, leading to acute toxicity, but provide a chaperone function that stabilizes proteins or membrane structure in surviving cells, allowing a robust recovery. This is further consistent with our heat shock findings, as preventing and resolving protein denaturation (e.g., through chaperone function) is critical for cell survival after heat shock; our data is consistent with TEAPs acting as macromolecular chaperones.

Several mechanisms of TEAP function have been hypothesized yet the inherent structurally disordered nature of TEAPs provides challenges for mechanism elucidation but likely plays a critical role in their dynamic capacity as conditions change during desiccation, however, some clues as to their mechanistic function do exist. Dsup expressed in HEK293 cells demonstrated specific transcription factor activation and gene expression patterns involved in DNA damage repair and antioxidant pathways in response to distinct stress stimuli (e.g., ROS and UV-C), suggesting Dsup acts through multiple distinct stress-specific protective mechanisms (e.g., shielding and regulation of gene expression)^50^. MAHS is hypothesized to transition from an intrinsically disordered protein to an amphipathic alpha-helical structure that can stabilize mitochondrial membranes in response to desiccation stress by preserving organization of polar phospholipid head groups in a manner similar to LEA proteins^9^. CAHS3 expressed in HEp-2 cells form filamentous networks in response to hyperosmotic stress that prevented loss of cell volume, increased cell elasticity, and enhanced viability, independent of the specific osmolytes present^70^. Similarly, HEK293 cells expressing CAHS D formed reversible cytoskeletal-like networks when treated with hyperosmotic stress that prevented cell volume decrease and suppressed metabolic activity^72^. Interestingly, recent studies of CAHS D demonstrated that reduced metabolic activity and not cell volume stability strongly correlated with increased survival post stress, suggesting that transient decreases in metabolic activity dependent on CAHS D condensate formation may be responsible for hyperosmotic stress tolerance and survival^51^. Further work is needed to elucidate the mechanisms of TEAP function in specific human cell populations.

This study has several important limitations. While these studies demonstrate that TEAPs can be heterologously expressed in human VECs, these studies were performed with an immortalized cell line; future work in primary cells and tissues is needed. Further, the primary endpoint of stress tolerance with MTT is not supported by independent quantitative measures of cell viability or proliferation (for example, LIVE/DEAD staining or EdU incorporation). MTT is a population-level measure of reductive activity, which correlates with cell population but can be impacted by changes in cellular metabolism^49,73^. With this assay, we cannot specifically attribute the results to changes in cell viability, metabolic function, cell proliferation, or other mechanisms. More detailed studies quantifying cell viability, cell proliferation, and stress pathway activation would enhance our understanding of the effects of TEAP expression. Lastly, this is not a mechanistic study, rather the purpose of this work is to demonstrate additional models of TEAP expression in human cells.

In conclusion, we demonstrated that AcGFP1-tagged TEAPs derived from the eutardigrade *R. varieornatus* can be stably expressed in routinely cultured human microvascular endothelial cells (HMEC1), TEAPs localize to specific cellular compartments in HMEC1 (e.g., cytosol, nucleus, and mitochondria) and do not show gross impacts on cell proliferation or morphology. We further tested if specific TEAPs (CAHS3, Dsup, MAHS, and LEAM) increase tolerance to osmotic and heat stressors. Notably, we show that protection against hyperosmotic stress only appears after a recovery period, and in contrast to prior work, show impairment during the acute hyperosmotic response. These data add to the existing spread of studies showing both positive and negative effects of TEAP expression and highlight the importance of constitutive transgene expression studies and careful selection of model systems for future applications.

## Supplementary Information

Below is the link to the electronic supplementary material.


Supplementary Material 1



Supplementary Material 2


## Data Availability

The data that was used for data shown in the figures are provided in the supplemental data.
