# Supplementary material for: Expression of tardigrade extremotolerance-associated proteins improves recovery but not acute stress response of human microvascular endothelial cells
Source: Sci Rep. 2026 Jun 16;16:27479. doi: 10.1038/s41598-026-57258-y (PMC13534718; doi:10.1038/s41598-026-57258-y)
Supplement: Supplementary file 1 [file 41598_2026_57258_MOESM2_ESM.docx]

**Supplementary Figures**

**Figure S1:** Expression of AcGFP1 (control) and AcGFP1-tagged TEAPs (CAHS3, Dsup, MAHS, and LEAM) in human microvascular endothelial cells, large field-of-view. Transgenic HMEC1 expressing AcGFP1-tagged TEAPs were stained live with the cell permeant cationic mitochondrial membrane dye TMRE (red) and nuclear DNA counterstain Hoechst (blue). AcGFP1 is expressed nonspecifically throughout the cell, CAHS3 localizes to the cytosol, Dsup localizes to the nucleus, and both MAHS and LEAM localize to the mitochondria.
